# Experiences of Everyday Ageism and the Health of Older US Adults

**DOI:** 10.1001/jamanetworkopen.2022.17240

**Published:** 2022-06-15

**Authors:** Julie Ober Allen, Erica Solway, Matthias Kirch, Dianne Singer, Jeffrey T. Kullgren, Valerie Moïse, Preeti N. Malani

**Affiliations:** 1 Department of Health and Exercise Science, University of Oklahoma, Norman; 2 Research Center for Group Dynamics, Institute for Social Research, University of Michigan, Ann Arbor; 3 Institute for Healthcare Policy and Innovation, University of Michigan, Ann Arbor; 4 Child Health Evaluation and Research Center, University of Michigan, Ann Arbor; 5 Center for Clinical Management Research, VA Ann Arbor Healthcare System, Ann Arbor, Michigan; 6 Department of Internal Medicine, University of Michigan, Ann Arbor; 7 Department of Health Management and Policy, University of Michigan, Ann Arbor

## Abstract

**Importance:**

Major incidents of ageism have been shown to be associated with poorer health and well-being among older adults. Less is known about routine types of age-based discrimination, prejudice, and stereotyping that older adults encounter in their day-to-day lives, known as everyday ageism.

**Objective:**

To examine the prevalence of everyday ageism, group differences and disparities, and associations of everyday ageism with indicators of poor physical and mental health.

**Design, Setting, and Participants:**

This cross-sectional study was conducted using survey data from the December 2019 National Poll on Healthy Aging among a nationally representative household sample of US adults ages 50 to 80 years. Data were analyzed from November 2021 through April 2022.

**Exposures:**

Experiences of everyday ageism were measured using the newly developed multidimensional Everyday Ageism Scale.

**Main Outcomes and Measures:**

Fair or poor physical health, number of chronic health conditions, fair or poor mental health, and depressive symptoms.

**Results:**

Among 2035 adults ages 50 to 80 years (1047 [54.2%] women; 192 Black [10.9%], 178 Hispanic [11.4%], and 1546 White [71.1%]; mean [SD] age, 62.6 [8.0] years [weighted statistics]), most participants (1915 adults [93.4%]) reported regularly experiencing 1 or more forms of everyday ageism. Internalized ageism was reported by 1664 adults (81.2%), ageist messages by 1394 adults (65.2%), and interpersonal ageism by 941 adults (44.9%). Mean Everyday Ageism Scale scores were higher for several sociodemographic groups, including adults ages 65 to 80 years vs those ages 50 to 64 years (11.23 [95% CI, 10.80-11.66] vs 9.55 [95% CI, 9.26-9.84]) and White (10.43 [95% CI, 10.20-10.67]; P < .001) and Hispanic (10.09 [95% CI, 9.31-10.86]; *P* = .04) adults vs Black adults (9.23 [95% CI, 8.42-10.03]). Higher levels of everyday ageism were associated with increased risk of all 4 negative physical and mental health outcomes examined in regression analyses (with odds ratios [ORs] per additional scale point as high as 1.20 [95% CI, 1.17-1.23] for depressive symptoms and *b* = 0.039 [95% CI, 0.029-0.048] for chronic health conditions; *P* values < .001). Internalized ageism was the category associated with the greatest increase in risk of poor outcomes for all health measures (with ORs per additional scale point as high as 1.62 [95% CI, 1.49-1.76] for depressive symptoms and *b* = 0.063 [95% CI, 0.034-0.092] for chronic health conditions; *P* values < .001).

**Conclusions and Relevance:**

This study found everyday ageism to be prevalent among US adults ages 50 to 80 years. These findings suggest that commonplace ageist messages, interactions, and beliefs may be harmful to health and that multilevel and multisector efforts may be required to reduce everyday ageism and promote positive beliefs, practices, and policies related to aging and older adults.

## Key Points

**Question:**

What is the prevalence of everyday ageism among US older adults, and is it associated with health?

**Findings:**

In this cross-sectional study of 2035 US adults ages 50 to 80 years, everyday ageism was prevalent (93.4%), experienced at differing levels by population sociodemographic characteristic, and associated with multiple indicators of poor physical and mental health.

**Meaning:**

These findings suggest that everyday ageism may warrant further attention and prioritization as a topic for additional research and as a preventable potential health hazard as people age.

## Teaser

This cross-sectional study investigates the prevalence of everyday ageism and its association with mental and physical health among adults ages 50 to 80 years.

## Introduction

Ageism is a common, socially condoned type of discrimination in the US.^1,2,3^ Ageism refers to stereotypes, prejudice, and discrimination related to old age, aging processes, and older adults. Major life events rooted in age-based discrimination have been associated with poor health outcomes.^4,5,6^ Less is known about routine ageism and whether it may also be associated with poorer health. Routine ageism affects more people and occurs more frequently, such as in comments about a “senior moment” or the barrage of antiaging commercials. These are examples of *everyday ageism*, defined as “brief verbal, nonverbal, and environmental indignities that convey hostility, a lack of value, or narrow stereotypes of older adults.”^7^ Everyday ageism is often subtle and may or may not be intentionally discriminatory. Nonetheless, these microaggressions may communicate that older adults are not fully accepted and respected, appreciated for their individuality, or deserving of the rights and privileges afforded other members of society.

Ageism and its associations with health are relatively understudied compared with other types of discrimination.^1^ A 2021 systematic review^8^ reported consistent evidence suggesting an association between ageism and adverse health outcomes. A noted limitation was that the examined studies lacked comprehensive ageism measures, instead assessing 1 or 2 types of intrapersonal ageism (eg, internalized beliefs and stereotypes). Experimental studies^9,10,11,12^ have found associations between examples of everyday ageism (eg, priming participants with negative, ageist stereotypes and ageist discrimination) and a variety of adverse health outcomes. Population-level survey research may augment this work by investigating the magnitude and generalizability of ageism as a potential health risk. Scales are needed that capture the multiple manifestations and mechanisms of ageism identified in the literature (eg, Iverson et al,^13^ Levy,^14^ and Swift et al^15^), including everyday ageism. Multidimensional scales are particularly important to evaluate the collective and potentially synergistic associations of ageism with health and to identify particularly harmful forms.

This study had 3 objectives: to examine the prevalence of everyday ageism among US adults ages 50 to 80 years using the newly developed, multidimensional Everyday Ageism Scale; explore disparities in everyday ageism; and investigate associations between everyday ageism and health. It builds on a brief report of preliminary findings^16^ by incorporating the Everyday Ageism Scale, which has subsequently been developed and documented as psychometrically sound,^7^ and comprehensively reporting study methods and findings. Everyday ageism was anticipated to be reported by an overwhelming majority of older US adults, consistent with previous ageism estimates from convenience samples of older adults^17^; more common among socially and economically disadvantaged groups; and associated with poor physical and mental health outcomes.

## Methods

This cross-sectional study was conducted in partnership with the University of Michigan National Poll on Healthy Aging (NPHA). The NPHA was deemed exempt from review and the requirement for informed consent waived by the University of Michigan Institution Review Board because data were deidentified. This study was also exempt and informed consent waived because studies of deidentified data are not classified as regulated human participant research under the Common Rule. This study followed the Strengthening the Reporting of Observational Studies in Epidemiology (STROBE) reporting guideline. The NPHA is a recurring, cross-sectional survey of US adults ages 50 to 80 years on health, health care, and healthy policy issues.^18^ Samples derive from the Ipsos KnowledgePanel, which is the largest nationally representative, probability-based online panel in the US.^19^ Ipsos recruits community-residing US residents identified with address-based sampling. NPHA samples are age stratified, divided equally between ages 50 to 64 years and 65 to 80 years. Poststratification weights reflect US Census population characteristics and differential participation rates; they factor in sex, age, race and ethnicity, language, education, income, home ownership, geographic variables, and nonresponse. Participants complete self-administered surveys online, with Ipsos providing web-enabled devices and free internet as needed.

This study is based on wave 6 of the NPHA, which was completed by 2048 older US adults in December 2019 and included questions on everyday ageism.^16^ The survey response rate was 2048 of 2664 individuals (76.9%). The analytic sample included 2035 participants and did not differ demographically from 13 excluded individuals missing data on their experiences with everyday ageism.

### Measures

#### Everyday Ageism

**Figure 1.**
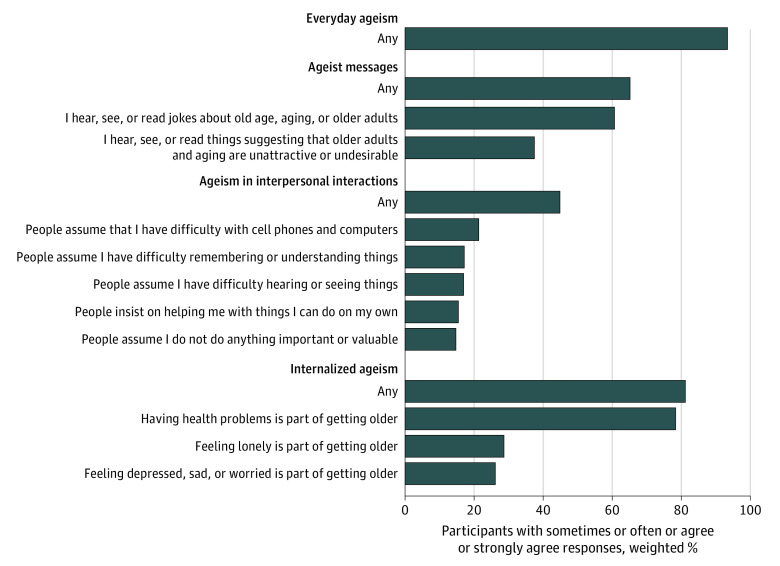
Prevalence of Any Experiences of Everyday Ageism

The Everyday Ageism Scale was used to assess the amount of routine ageism participants reported experiencing in their daily lives.^7^ The scale captures a phenomenon similar to everyday discrimination^20^ but emphasizes age-specific discrimination. Items ask about easily identifiable beliefs, experiences, and concrete behaviors representing commonly encountered examples of everyday ageism but do not require respondents identify them as such. The scale has 10 items and 3 dimensions (Figure 1): frequency of exposure to *ageist messages* in the form of environmental and social cues reflecting ageist prejudices and stereotypes (2 items); frequency of *ageism in interpersonal interactions*, specifically being targeted by discrimination rooted in others’ assumptions and stereotypes about older adults (5 items); and endorsement of *internalized ageism*, reflecting individually held beliefs linking aging and health (3 items). The scale has been shown to be psychometrically sound and appropriate for use as a single scale or as a set of 3 subscales.^7^ Scores were calculated by summing responses (4-point ordinal options of frequency or agreement) for participants completing at least 9 of 10 items. Higher scale scores indicated more everyday ageism, with a potential range of 0 to 30. Cronbach α was 0.761.

#### Sociodemographic Characteristics

Sociodemographic characteristics included age in years, age group (50-64 or 65-80 years), sex (man or woman), self-identified race and ethnicity (categories were Hispanic, non-Hispanic Black, non-Hispanic White, and other racial categories), married or living with a partner (yes or no); education (≤high school diploma, some college, or ≥bachelor’s degree), annual household income (21 income ranges), employed (yes or no), metropolitan area (yes or no), region (Midwest, Northeast, South, or West), and media use indicating mean hours spent viewing television, the internet, or magazines daily (>4, 2-4, or <2). For race, participants were asked to select all that applied: American Indian or Alaska Native, Asian, Black or African American, Native Hawaiian or Pacific Islander, White, or a different race; options for ethnicity were Hispanic or Non-Hispanic. Race and ethnicity were asked in separate questions. These were recoded to reflect the largest racial and ethnic categories used in health disparities research in the US: all those identifying as Hispanic (any race); non-Hispanic Black, non-Hispanic White, and all other racial categories, including those identifying with more than 1 racial group. There were no missing sociodemographic data, with the exception of media use (6 individuals were omitted from regression analyses).

#### Health Outcomes

We examined 2 general indicators of physical health and 2 of mental health. *Fair or poor physical health* indicated responses of fair or poor to a single item: “In general, how would you rate your physical health?” (reference group: good or better). *Chronic health conditions* reflected the raw number of diagnosed chronic health conditions among 9 conditions: hypertension, high cholesterol, heart disease or attack, stroke, diabetes or prediabetes, cancer, chronic lower respiratory disease, osteoarthritis or joint problem, and chronic pain. *Fair or poor mental health* indicated responses of fair or poor to a single item: “In general, how would you rate your mental health?” (reference group: good or better). *Depressive symptoms* signified report of some depressive symptoms at least several days during the prior 2 weeks (reference group: no symptoms). We used the 2-item Patient Health Questionnaire^21^ (PHQ-2), which asked participants if they were bothered by little interest or pleasure in doing things or by feeling down, depressed, or hopeless.

### Statistical Analysis

Analyses were completed with Stata statistical software version 17.0 (StataCorp), poststratification weights, and 2-tailed significance tests with *P* < .05. Prevalence of everyday ageism was examined in 2 ways. First, any experiences with everyday ageism was assessed. This was indicated by often or sometimes or strongly agree or agree responses to any items in the full Everyday Ageism Scale. Any experiences were also determined for each of the 3 scale categories (ie, subscale dimensions) and specific forms of everyday ageism (ie, individual scale items). Second, aggregate scale scores were used to assess the amount of everyday ageism overall (full scale), by category, and by specific form. Differences and disparities in amount of everyday ageism were identified by comparing mean everyday ageism scores by sociodemographic group using bivariate statistical tests that allow weighted estimation: linear regression for dichotomous grouping variables and analysis of variance for multiple group comparisons with pairwise margin comparisons.

To examine associations between amount of everyday ageism and health, each health outcome (dependent variable) was regressed on everyday ageism scale scores (independent variable) in separate models before and after adjusting for sociodemographic characteristics (dummy coded except for age and income). Logistic regression (for fair or poor physical and mental health and depressive symptoms) and negative binominal regression (for number of chronic health conditions, see histogram in eFigure in the Supplement) were used to estimate multivariate models and marginal means with 95% CIs for graphing. Post hoc analyses were used to investigate which everyday ageism categories and forms were associated with the greatest difference in identified outcomes. This entailed replicating the models while first replacing the full scale with 3 category scale scores and next replacing it with 10 individual items.

## Results

The sample comprised 2035 US adults (1047 [54.2%] women; 178 Hispanic [11.4%], 192 non-Hispanic Black [10.9%], 1546 non-Hispanic White [71.1%], and 119 identifying other racial categories [6.7%]; mean [SD] age, 62.6 [8.0] years). Participant characteristics (Table 1) are weighted statistics and were generally comparable with those of this age group nationwide, as expected given the weighting strategy, with the exception of a median household income that was greater than the national median. Within the sample, 327 participants (17.7%) rated their physical health as fair or poor and 1707 participants (82.3%) rated their physical health as good or better, while 124 participants (7.2%) rated their mental health as fair or poor and 620 participants (32.3%) reported depressive symptoms. Participants had a mean (SD) of 1.57 (1.49) chronic health conditions.

**Table 1.** Participant Characteristics

Characteristic	Participants, No. (weighted %) (N = 2035)
Age, weighted mean (SD), y	62.60 (8.04)
Age group, y	
65-80	1034 (39.7)
50-64	1001 (60.3)
Sex	
Women	1047 (52.4)
Men	988 (47.6)
Race and ethnicity	
Hispanic	178 (11.4)
Non-Hispanic Black	192 (10.9)
Non-Hispanic White	1546 (71.1)
Other racial categories^a^	119 (6.7)
Married or living with partner	
No	632 (33.2)
Yes	1403 (66.8)
Education	
≤High school diploma	691 (39.8)
Some college	602 (26.7)
≥Bachelor’s degree	742 (33.4)
Annual household income range, weighted median, $	60 000-74 999
Employed	
No	1065 (48.8)
Yes	970 (51.2)
Metro area	
No	317 (15.2)
Yes	1718 (84.8)
Region	
Midwest	460 (21.2)
South	744 (38.1)
West	458 (22.9)
Northeast	373 (17.8)
Media use, h/d	
>4	637 (31.4)
2-4	764 (37.8)
<2	598 (30.8)
Health outcome	
Fair or poor physical health	327 (17.7)
Chronic health conditions, No. (0-9), weighted mean (SD)	1.57 (1.49)
Fair or poor mental health	124 (7.2)
Depressive symptoms	620 (32.3)

^a^ Includes individuals identifying as non-Hispanic and American Indian, Alaska Native, Asian, Native Hawaiian, Pacific Islander, multiple races, or a different race.

### Prevalence and Forms of Everyday Ageism Reported by Older Adults

**Figure 2.**
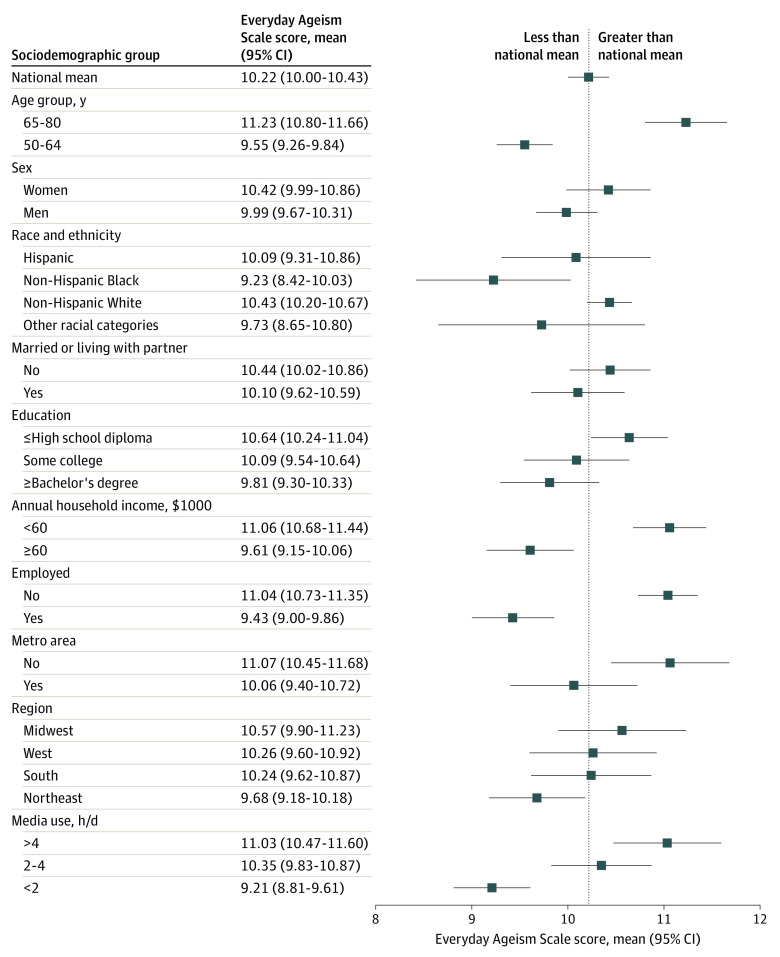
Everyday Ageism by Sociodemographic Group

A total of 1915 adults (93.4%) ages 50 to 80 years reported regularly experiencing at least 1 of 10 forms of everyday ageism (Figure 1). Internalized ageism was the most commonly endorsed category (1664 adults [81.2%]), followed by exposure to ageist messages (1394 adults [65.2%]) and ageism in interpersonal interactions (941 adults [44.9%]). The mean (SD) amount of everyday ageism, as indicated by Everyday Ageism Scale score, in this nationally representative sample was 10.22 (95% CI, 10.00-10.43) and ranged from 0 to 27 (Figure 2).

### Differences and Disparities in Everyday Ageism

Adults ages 65 to 80 years reported a larger mean amount of everyday ageism than those ages 50 to 64 years (11.23 [95% CI, 10.80-11.66] vs 9.55 [95% CI, 9.26-9.84]; *P* < .001), and women reported more than men (10.42 [95% CI, 9.99-10.86] vs 9.99 [95% CI, 9.67-9.84]; *P* = .05), although this difference was not statistically significant (Figure 2). Everyday ageism varied by race and ethnicity; compared with non-Hispanic Black adults (9.23 [95% CI, 8.42-10.03], non-Hispanic White adults 10.43 [95% CI, 10.20-10.67]; *P* < .001) and Hispanic adults (10.09 [95% CI, 9.31-10.86]; *P* = .04) reported higher mean everyday ageism scores. Everyday ageism differed by indicators of lower socioeconomic status (ie, education, income, and employment). Adults in rural areas (11.07 [95% CI, 10.45-11.68]) reported more everyday ageism than those in metropolitan areas (10.06 [95% CI, 9.40-10.72]; *P* = .003), as did those in the Midwest (10.57 [95% CI, 9.90-11.23]) compared with those in the Northeast (9.68 [95% CI, 9.18-10.18; *P* = .006). Adults spending more than 4 hours daily on media reported more everyday ageism (11.03 [95% CI, 10.47-11.60]) than those with less media use (2-4 hours: 10.35 [95% CI, 9.83-10.87]; *P* = .004; <2 hours: 9.21 [95% CI, 8.81-9.61]; *P* < .001).

### Everyday Ageism and Health

**Figure 3.**
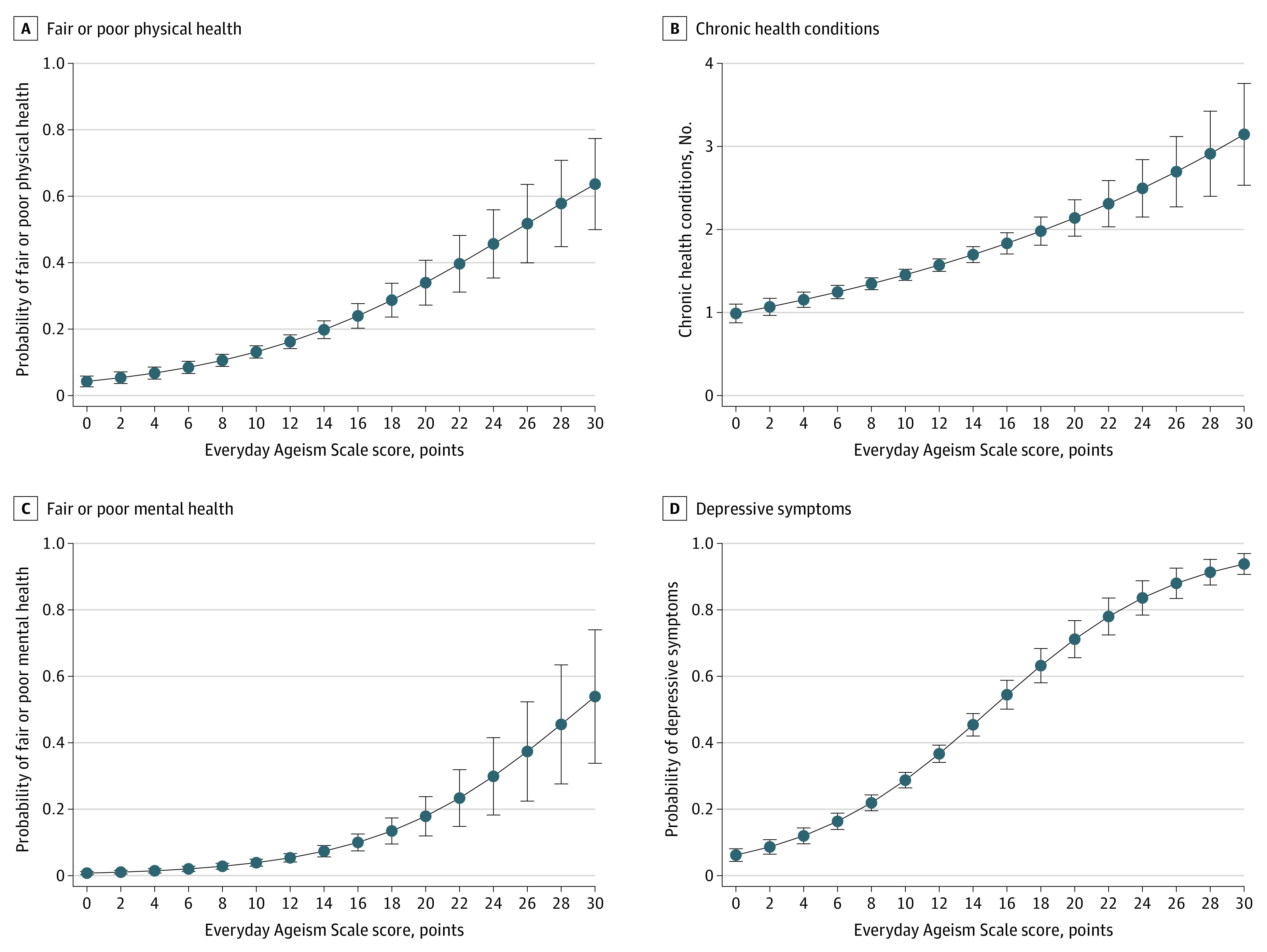
Associations Between Everyday Ageism and Health Outcomes Outcomes are adjusted for age, sex, race and ethnicity, married or living with partner status, education level, household income level, employment status, metro area, region, and daily media use.

Everyday ageism was associated with poor physical and mental health across all 4 outcomes examined (Table 2 and Figure 3). For each additional point on the Everyday Ageism Scale, odds of fair or poor physical health increased by 1.13-fold (95% CI, 1.01-1.17; *P* < .001) after adjusting for sociodemographic characteristics. The probability of fair or poor physical health was 0.082 for adults reporting everyday ageism 1 SD below the mean. This increased to 0.134 (63.4%) for those reporting mean levels of everyday ageism and 0.213 for those 1 SD above the mean (for an increase in probability of 59.0% vs the mean) (Table 2 and Figure 3A). Everyday ageism was associated with number of chronic health conditions (*b* = 0.039 [95% CI, 0.029-0.048]; *P* < .001): 1.23 conditions at 1 SD below the mean everyday ageism scale score, 1.47 conditions at the mean (for an increase of 19.5%), and 1.75 conditions at 1 SD above the mean (for an additional increase of 19.5%) (Table 2 and Figure 3B). Odds of fair or poor mental health and depressive symptoms increased by factors of 1.18 (95% CI, 1.13-1.24; *P* < .001) and 1.20 (95% CI, 1.17-1.23; *P* < .001), respectively, with each additional point on the Everyday Ageism Scale. Probabilities of fair or poor mental health were low but increased by more than 2-fold with each SD increase in ageism score (ie, increases of 107.5%-110.5%) (Figure 3C). Probabilities of depressive symptoms were higher and increased 65.4% to 90.3% with each SD (Table 2 and Figure 3D).

**Table 2.** Associations Between Everyday Ageism and Health Outcomes^a^

	Fair or poor physical health (n = 2028)		Chronic health conditions, No. (n = 1917)		Fair or poor mental health (n = 2024)			Depressive symptoms (n = 2028)	
	Result	*P* value	Result	*P* value	Result		*P* value	Result	*P* value
Per 1 point on Everyday Ageism Scale	1.130 (1.095-1.166)^b^	<.001	0.039 (0.029-0.048)^c^	<.001	1.183 (1.131-1.238)^b^	<.001		1.199 (1.166-1.233)^b^	<.001
Level^d^									
1 SD <mean	0.082^e^	NA	1.23^f^	NA	0.019^e^	NA		0.155^e^	NA
Mean	0.134^e^	NA	1.47^f^	NA	0.040^e^	NA		0.295^e^	NA
1 SD > mean	0.213^e^	NA	1.75^f^	NA	0.083^e^	NA		0.488^e^	NA
Model	195.98^g^	<.001	344.42^g^	<.001	133.79^g^	<.001		226.56^g^	<.001

Abbreviations: NA, not applicable; OR, odds ratio.

^a^ Adjusted for age, sex, race and ethnicity, married or living with partner status, education level, household income level, employment status, metro area, region, and daily media use.

^b^ Values are ORs with 95% CIs.

^c^ Value is *b* with 95% CI.

^d^ Covariates held at mean values.

^e^ Values are probabilities.

^f^ Values are No.

^g^ Values are χ^2^.

Post hoc analyses were used to investigate everyday ageism categories and forms associated with the greatest increases in risk of poor health outcomes (eTables 1 and 2 in the Supplement). Internalized ageism was associated with the greatest increases in risk for all 4 health outcomes (ORs ranging from 1.34 [95% CI, 1.23-1.46] for fair or poor physical health to 1.62 [95% CI, 1.49-1.76] for depressive symptoms and *b* = 0.063 [95% CI, 0.034-0.092] for chronic health conditions; all *P* < .001), followed by interpersonal ageism (ORs ranging from 1.13 [95% CI, 1.09-1.18]; *P* < .001 for depressive symptoms to 1.17 [95% CI, 1.09-1.26]; *P* < .001 for fair or poor mental health and *b* = 0.025 [95% CI, 0.008-0.041]; *P* = .003 for chronic health conditions) (eTable 1 in the Supplement). More frequent exposure to ageist messages was associated with lower probability of fair or poor physical health (OR, 0.88 [95% CI, 0.79-0.98]; *P* = .02) and more chronic health conditions (*b* = 0.048 [95% CI, 0.013-0.084]; *P* = .007) but was not associated with mental health indicators. Individual ageism items associated with physical and mental health outcomes were generally associated with increases in risk of negative outcomes (eTable 2 in the Supplement). For example, endorsement of the belief that health problems were part of getting older was the item associated with the largest increase in odds of fair or poor physical health (OR, 1.93 [95% CI, 1.49-2.51]; *P* < .001); it was not associated with other health outcomes. Endorsement of the belief that feeling depressed, sad, or worried is part of getting older was the item associated with the greatest increase in number of chronic health conditions (*b* = 0.112 [95% CI, 0.047-0.177]; *P* = .001) and odds of poor mental health outcomes (fair or poor mental health: OR, 2.39 [95% CI, 1.70-3.35]; *P*  <.001; depressive symptoms: 3.16 [95% CI, 2.63-3.81]; *P* < .001); it was the item associated with the second highest increase in odds of fair or poor physical health (OR, 1.45 [95% CI, 1.18-1.78]; *P* < .001). Reports that people assumed participants had difficulty hearing or seeing things were associated with all 4 outcomes (eTable 2 in the Supplement). Other associations included unnecessary help (associated with fair or poor physical health, more chronic health conditions, and depressive symptoms), exposure to ageist jokes (associated with depressive symptoms), and assumptions of cell phone or computer difficulties (associated fair or poor physical health) (eTable 2 in the Supplement). Assumptions of memory or comprehension difficulties were associated with a higher probability of fair or poor mental health (OR, 1.03 [95% CI, 1.00-1.05]; *P* = .03) but a lower probability of depressive symptoms (OR, 0.98 [95% CI, 0.97-0.99]; *P* = .001).

## Discussion

This cross-sectional study documented the pervasiveness of everyday ageism and its associations with poor health among older US adults using the newly developed, multidimensional Everyday Ageism Scale.^7^ More than 9 of 10 adults ages 50 to 80 years in the nationally representative NPHA sample reported experiencing 1 or more forms of everyday ageism regularly. This was generally consistent with previous ageism prevalence rates (77%-91%) derived from other ageism measures and convenience samples of older North American adults.^17^

Previous findings on ageism differences and disparities have been inconsistent.^22^ This study identified disparities in everyday ageism by age and socioeconomic status. The patterning was consistent with social stratification in the US in which populations are multiply marginalized at intersections of their identities (eg, being an older adult and low income).^23^ Documented differences by race and ethnicity were opposite the typical patterning of social disadvantages, although not without precedent in ageism research.^24^ Identified differences likely reflected racial and ethnic variations in perceptions of everyday ageism rather than exposure.^25^ Ageism may be the first major type of discrimination some White adults experience, which may increase their awareness compared with other racial and ethnic groups more habituated to discrimination. Given the centrality of race and ethnicity in the lives of members of racial and ethnic minority groups, they may attribute discrimination to their race or ethnicity rather than their age. Research on more objective examples of ageism (eg, employment discrimination) supports the premise that older adults who are members of racial and ethnic minorities experience more ageism.^25,26^ More research is needed to investigate how everyday ageism may be associated with health disparities within the older adult population and whether social identities moderate associations between everyday ageism and health.

Everyday ageism was associated with all 4 health outcomes examined, including 2 indicators each for physical and mental health. Odds of negative health outcomes increased 59.0% to 110.5% with 1 SD increase in everyday ageism. The associated number of chronic health conditions also increased, albeit less markedly. Although this study could not determine whether experiences with everyday ageism preceded the development of poor health or vice versa, empirical research suggests that ageism is associated with greater changes in health than the converse.^27,28^

Everyday ageism may affect health outcomes via multiple pathways. Ageism may hamper quality of older adults’ interactions with health care clinicians. Ageist cues, beliefs, and interpersonal interactions may serve as stereotype threats, primes for stereotype embodiment, and models of normative expectations for older adults, all of which have been associated with poor health outcomes.^9,10,12,14^ Accordingly, everyday ageism may be a chronic stressor in the lives of older adults. Researchers posit that exposure to chronic stressors repeatedly activates psychological, cognitive, behavioral, and biological stress responses, resulting in accelerated aging and increased risk for chronic disease and premature mortality.^1,29,30,31,32^ Inverse associations are also plausible. Older adults with poor health may experience more ageist messages and discrimination (and discrimination based on health and disability) and personally relevant evidence supporting negative beliefs associating age with health.

Internalized ageism was the category most commonly endorsed in our study (81.2% of participants) and associated with the largest increases in risk for all health outcomes. This provides further evidence suggesting the importance of this dimension of ageism, which has been most frequently investigated in relation to health.^8,12,14^ The item stating that “having health problems is part of getting older” is worthy of comment given its high endorsement rate and questions about whether the item captured ageism or immutable outcomes of chronological aging. Associating poor health with old age may be the most deeply rooted aging stereotype, despite evidence to the contrary (for example, 82.3% of participants in the current study rated their physical health as good or better). Several issues may contribute to the potency of this stereotype. Physiological and cognitive changes accompanying old age are often characterized negatively as “problems” or “deterioration,” rather than viewed neutrally as part of human development.^33^ A growing body of research implicates ageism in poor health outcomes.^1,8,9,14^ Disentangling health outcomes attributable to chronological aging from preventable health outcomes attributable to the social construct of ageism is a challenge for future research. Altering societal attitudes associating poor health and aging may prove even more difficult.

Frequent ageism in interpersonal interactions was less commonly reported (44.9%) but also associated with all negative health outcomes. Exposure to ageist messages, while common (65.2%), was the only category exhibiting mixed associations with the health outcomes. Because ageist messages may shape individual and societal beliefs about aging and older adults,^14,34^ it is plausible that ageist messages may be associated with health indirectly. Collectively, our findings suggest that all 3 categories of everyday ageism should be considered potentially associated with detrimental health outcomes.

Study results may inform intervention efforts to reduce potential health harms associated with ageism. Frequent exposure to commonplace ageist messages, interactions, and beliefs often perceived as trivial may be more harmful than is generally recognized. Internal and external sources of ageism may have ramifications for health. Taken together, our findings suggest that multilevel and multisector interventions may be most effective at reducing age-based discrimination and promoting more positive, nuanced views of aging.

### Limitations

This study has several limitations. Responses to the Everyday Ageism Scale may be affected by recall bias, social desirability, misattribution, and a lack of awareness, which may result in an underestimate of the prevalence of everyday ageism. Results may not generalize to groups excluded from the NPHA sample, such as adults in institutions or prisons, ages older than 80 years, or unable to complete surveys online. Temporality and causality could not be investigated owing to the cross-sectional nature of the NPHA.

## Conclusions

This study documented the ubiquity of an understudied type of ageism, everyday ageism, among US adults ages 50 to 80 years. We found that commonplace ageist messages, interactions, and beliefs were associated with negative health outcomes. These findings suggest that multilevel and multisector efforts may be required to reduce everyday ageism and promote positive beliefs, practices, and policies related to aging and older adults. This research raises the question of whether aging-related health problems reflect associations of ageism with adverse outcomes, thereby presenting antiageism efforts as a strategy for promoting older adult health and well-being.

## Notes

Supplement. **eFigure.** Histogram for Number of Chronic Conditions **eTable 1.** Associations Between Everyday Ageism Categories and Health Outcomes **eTable 2.** Associations Between Individual Everyday Ageism Items and Health Outcomes
