# Supplementary material for: Experiences of Everyday Ageism and the Health of Older US Adults
Source: JAMA Netw Open. 2022 Jun 15;5(6):e2217240. doi: 10.1001/jamanetworkopen.2022.17240 (PMC9201677; doi:10.1001/jamanetworkopen.2022.17240)
Supplement: Supplement. — eFigure. Histogram for Number of Chronic Conditions eTable 1. Associations Between Everyday Ageism Categories and Health Outcomes eTable 2. Associations Between Individual Everyday Ageism Items and Health Outcomes [file jamanetwopen-e2217240-s001.pdf]

Supplemental Online Content

Allen JO, Solway E, Kirch M, et al. Experiences of everyday ageism and the health of older US adults. *JAMA Netw Open*. 2022;5(6):e2217240. doi:10.1001/jamanetworkopen.2022.17240

eFigure. Histogram for Number of Chronic Conditions

eTable 1. Associations Between Everyday Ageism Categories and Health Outcomes

eTable 2. Associations Between Individual Everyday Ageism Items and Health Outcomes

This supplemental material has been provided by the authors to give readers additional information about their work.

© 2022 Allen JO et al. *JAMA Network Open.*

eFigure. Histogram for Number of Chronic Conditions

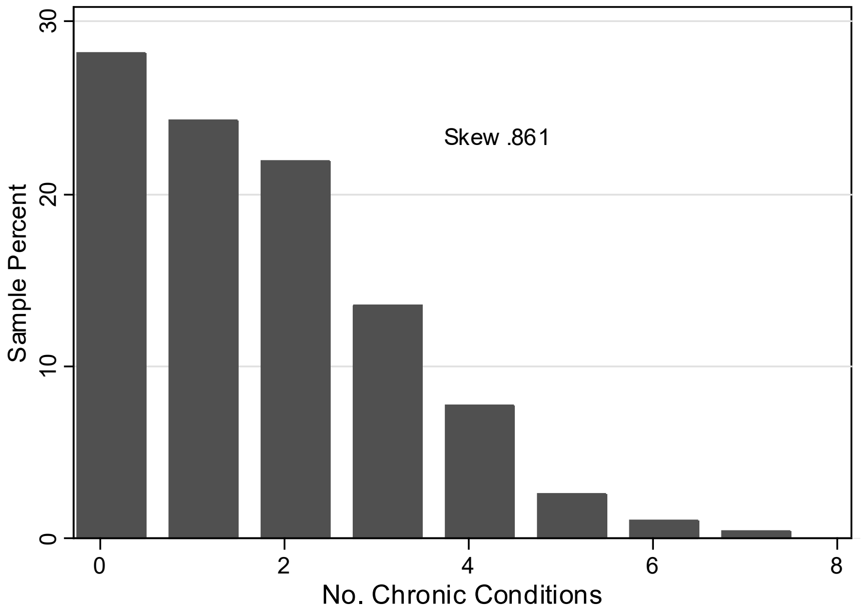

© 2022 Allen JO et al. *JAMA Network Open.*

eTable 1. Associations Between Everyday Ageism Categories and Health Outcomes[a]

Categories	**Fair or poor physical health** (n = 2028)		**No. chronic health conditions** (n = 1917)		**Fair or poor mental health** (n = 2024)		**Depressive symptoms** (n = 2028)	
	OR (95% CI)	*P* value	*b* (95% CI)	*P* value	OR (95% CI)	*P* value	OR (95% CI)	*P* value
Ageist Messages	.875 [.785-.977]	**.02**	.048 [.013-.084]	**.007**	.965 [.827-1.126]	.65	1.052 [.962-1.150]	.27
Interpersonal Ageism	1.163 [1.105-1.224]	**<.001**	.025 [.008-.041]	**.003**	1.170 [1.087-1.260]	**<.001**	1.134 [1.086-1.183]	**<.001**
Internalized Ageism	1.338 [1.228-1.456]	**<.001**	.063 [.034-.092]	**<.001**	1.477 [1.301-1.677]	**<.001**	1.617 [1.485-1.761]	**<.001**
Model X^2 *p*	210.50	**<.001**	310.70	**<.001**	138.99	**<.001**	256.43	**<.001**

Abbreviation: OR, odds ratio.

[a] Adjusted for age, sex, race and ethnicity, married or living with partner status, education level, household income level, employment status, metro area, region, and daily media use.

$P \leq .05$ emphasized with bold text

© 2022 Allen JO et al. *JAMA Network Open.*

eTable 2. Associations Between Individual Everyday Ageism Items and Health Outcomes[a]

Individual Everyday Ageism Items	**Fair or poor physical health** (n = 2028)		**No. chronic health conditions** (n = 1917)		**Fair or poor mental health** (n = 2024)		**Depressive symptoms** (n = 2028)	
	OR (95% CI)	*P* value	*b* (95% CI)	*P* value	OR (95% CI)	*P* value	OR (95% CI)	*P* value
I hear, see, and/or read jokes about old age, aging, or older people	.852 [.686-1.058]	.15	.003 [-.007-.012]	.55	.798 [.587-1.084]	.15	.959 [.895-1.278]	.24
I hear, see, and/or read things suggesting that older adults and aging are unattractive or undesirable	.893 [.719-1.110]	.31	.047 [-.007-.102]	.09	1.214 [.895-1.646]	.21	1.173 [1.007-1.366]	**.04**
People assume that I have difficulty with cell phones and computers	1.036 [1.014-1.059]	**.001**	-.000 [-.006-.005]	.93	1.001 [.973-1.030]	.94	1.028 [.992-1.066]	.13
People assume I have difficulty remembering and/or understanding things	1.010 [.987-1.033]	.40	-.001 [-.007-.005]	.74	1.025 [1.003-1.0467]	**.03**	.982 [.971-.992]	**.001**
People assume I have difficulty hearing and/or seeing things	1.236 [1.032-1.479]	**.02**	.058 [.004-.112]	**.04**	1.502 [1.171-1.927]	**.001**	1.172 [1.009-1.361]	**.04**
People insist on helping me with things I can do on my own	1.660 [1.361-2.025]	**<.001**	.084 [.025-.144]	**.006**	1.047 [.781-1.403]	.76	1.241 [1.047-1.471]	**.01**
People assume I do not do anything important or valuable	1.006 [.989-1.024]	.47	-.005 [-.014-.004]	.28	.995 [.970-1.020]	.67	.992 [.977-1.007]	.28
Having health problems is part of getting older	1.934 [1.488-2.514]	**<.001**	.003 [-.005-.011]	.47	1.277 [.890-1.833]	.18	1.187 [.960-1.468]	.11
Feeling lonely is part of getting older	.987 [.965-1.008]	.22	.006 [-.001-.013]	.08	1.000 [.977-1.024]	.97	1.007 [.985-1.030]	.52
Feeling depressed, sad, or worried is part of getting older	1.449 [1.177-1.783]	**<.001**	.112 [.047-.177]	**.001**	2.386 [1.700-3.349]	**<.001**	3.162 [2.627-3.805]	**<.001**
Model X^2, *p*	211.39	**<.001**	340.50	**<.001**	144.33	**<.001**	295.70	**<.001**

Abbreviation: OR, odds ratio.

[a] Adjusted for age, sex, race and ethnicity, married or living with partner status, education level, household income level, employment status, metro area, region, and daily media use. *P*≤.05 emphasized with bold text

© 2022 Allen JO et al. *JAMA Network Open.*
